# Mortality of Patients With Severe COVID-19 in the Intensive Care Unit: An Observational Study From a Major COVID-19 Receiving Hospital

**DOI:** 10.7759/cureus.10906

**Published:** 2020-10-12

**Authors:** Fawad Rahim, Said Amin, Mohammad Noor, Sher Bahadur, Huma Gul, Afsheen Mahmood, Muhammad Usman, Muhammad Asif Khan, Raza Ullah, Khalid Shahab

**Affiliations:** 1 Internal Medicine, Khyber Girls Medical College, Peshawar, PAK; 2 Internal Medicine, Hayatabad Medical Complex Peshawar, Peshawar, PAK; 3 Epidemiology and Public Health, Khyber Institute of Child Health, Peshawar, PAK; 4 Critical Care Medicine, Hayatabad Medical Complex Peshawar, Peshawar, PAK

**Keywords:** covid-19, mortality, intensive care, survival, comorbidities

## Abstract

Objective

To determine the mortality of patients with severe COVID-19 in the intensive care unit (ICU) in relation to age, gender, co-morbidities, ventilatory status, and length of stay (LOS).

Methods

This was a cross-sectional study based on data retrieved for 204 patients admitted to the ICU of Hayatabad Medical Complex, Peshawar, Pakistan, from April to August 2020. Study variables were age, gender, co-morbid conditions, ventilatory status, and length of stay (LOS). The data were analyzed using SPSS version 21 (IBM Corp., Armonk, NY). The independent t-test and the chi-square test were used to compare the means and frequencies of variables. Multivariate regression analysis was used to predict the likelihood of mortality.

Results

The overall mortality was 77%. Non-invasive ventilation (NIV) was administered to 61.8% of patients. Mortality was higher for invasive mechanical ventilation (IMV) (93.6% vs 66.7%, p<0.001) and for over 60 years (87.3% vs 72.3%, p=0.019). Mortality without co-morbidities was 75.2%. Comparative mortality rates for at least one co-morbidity (79.7%), diabetes mellitus (80.0%), hypertension (100%), diabetes mellitus and hypertension both (87.1%), and chronic obstructive pulmonary disease (75%) were insignificant. The LOS for survivors was longer (8.9±8.9 versus 5.4±5.2 days, p=0.017). The LOS < 24h was associated with higher mortality (85.9% vs 72.9%, p=0.040). On multivariable regression, the likelihood of mortality was high for IMV (7.330, 95% CI 2.667 - 20.143, p<0.001) and elderly (>60 years) patients (2.607, 95%CI 1.063 - 6.394, p=0.036). Mortality decreased with LOS longer than 24h (0.412, 95%CI 0.173 - 0.982, p=0.045). Co-morbidities did not have any effect on mortality.

Conclusions

Age more than 60 years and IMV were independent risk factors for higher mortality. Longer ICU stay, specifically more than 24 hours, was associated with lower mortality but LOS less than 24 hours might not have a causal relationship with mortality. The odds of survival were not affected by co-morbidities.

## Introduction

Coronavirus disease 2019 (COVID-19) started as atypical pneumonia in Wuhan, China, in December 2019 and soon became a global pandemic [1]. Globally, there are 28,285,700 confirmed cases as of September 12, 2020, and 911,255 have succumbed to the disease [2]. Pakistan reported its first case on February 26, 2020, and as of September 12, 2020, there are 300,955 confirmed cases with a death tally of 6,373 [3]. The clinical manifestations of COVID-19 vary from asymptomatic seroconversion to mild upper respiratory tract infection, severe pneumonia, and multiorgan failure [1].

The mortality of COVID-19 pneumonia is higher than other viral pneumonia [4]. After the outbreak, a large number of patients requiring respiratory support placed an unprecedented demand for intensive care services [5]. This necessitated a rapid expansion of intensive care infrastructure, capacity building, and staffing in many countries [6]. The data in terms of mortality, ventilatory support, comorbid conditions, and length of hospital stay is conflicting because different authors have reported the outcomes of a fraction of admitted patients and at variable durations since admission [7-8].

Multiple factors influence ICU mortality in COVID-19. These include the severity of the illness itself, gender, ethnicity, age, comorbid conditions, blood type, mode of ventilatory support, in addition to the availability of trained staff in the ICU [9]. Studies have reported nearly 100% mortality amongst patients on invasive mechanical ventilation (IMV) during the peak of the pandemic [10]. Mortality among patients on IMV was 88.6%, 97%, 43%, 31%, 88.8%, 22%, and 40%-60% in New York [7], China [11], UK [12], Spain [13], Australia [14], and India [15], respectively. With time, the mortality in ICUs declined to around 40% [4]. In the early days of the outbreak, there were no international guidelines for managing patients admitted to the ICU, and the rationing of resources based on local policies in overwhelmed ICUs might have added to divergent data. It is likely that due to the pressure of the pandemic on ICU services, there has been widespread use of other respiratory support (NIV or high-flow nasal oxygen) outside ICUs, and, therefore, patients admitted to the ICU were disproportionately sicker.

Fatality rates vary significantly by country, as the magnitude and velocity of surge fluctuated in different regions of the world on different time frames [7-8,10]. Moreover, the preparedness of the health services, the response of the community, and cultural and religious beliefs might have added to the outcome [16]. Pakistan is a developing country where one-third of the population is living below the poverty line (less than one dollar a day) [17]. The health system is weak and already overstretched. COVID-19 further added to this. There was a social stigma attached to a COVID-19 diagnosis [18]. Patients would often hide epidemiological risk factors & even clinical features of the disease to avoid being diagnosed. Many patients would not come early even with severe disease to the hospital and relatives would keep them at home till very late. Moreover, the shortage of ICU beds, ventilators, and trained staff was another worry, adding to the nightmare of the worst outcome for the patients of COVID-19 in Pakistan [19].

There was no harmony in the vision and actions of different stakeholders and policymakers of the government to prevent the spread of the virus [20]. Amidst the pandemic and prevailing ground realities, the general perception of the medical community was that Pakistan’s overall mortality, in general, and ICU mortality, in particular, will be strikingly different from the rest of the world [21]. This necessitated determining the survival of COVID-19 patients in relation to comorbid conditions and ventilatory support in our population.

## Materials and methods

This cross-sectional study was carried out in a major COVID-19 receiving tertiary care hospital in Peshawar, Pakistan. It is a 1200-bedded hospital, caring for patients coming from all over the province. Since there were no national or international guidelines for treating severely ill patients with COVID-19 in the early days of the pandemic, a local criterion for admission to the ICU was devised. While devising the criteria, the severity of the disease itself, the degree of hypoxia, hemodynamic stability, co-morbid conditions, and multiorgan involvement were considered. The study was approved by the institutional review board of Khyber Girls Medical College/Hayatabad Medical Complex, Peshawar, Pakistan. Permission was obtained from the medical director. Data were retrieved for 204 patients admitted from April 1, 2020, till August 31, 2020. Study variables were age, gender, comorbidities, ventilatory status, length of stay (LOS), and outcomes in terms of survival and death. Ventilatory status was taken as the highest level of respiratory support received, either non-invasive ventilation (NIV) (continuous positive airway pressure therapy (CPAP) or bilevel positive airway pressure (BiPAP)) or IMV. Survival was defined as shifting from the ICU to a general isolation ward when the patient improved clinically and did not need further ICU care. The data were analyzed using SPSS version 21. The independent t-test and chi-square test were used to determine the statistical significance of differences in age, gender, comorbidities, ventilatory status, and LOS between survivors and non-survivors. The multivariable regression model was used to predict the likelihood of mortality for the above-mentioned variables.

## Results

The mean age of the study population was 55.7 ± 11.6 years. Out of the total of 204 patients, 151 (74%) were male. Around two-thirds of patients (69.1%) were under the age of 60 years. One-hundred twenty-five (61.3%) patients did not have any co-morbidity. The most common co-morbidities were diabetes mellitus (DM) and hypertension (HTN) (15.2% of patients had both DM and HTN in addition to 12.3% who were diabetic only). The mean LOS in ICU was 6.2 ± 6.5 days, and 64 (31.4%) patients spent less than 24 hours in the ICU. Demographic parameters are summarized in Table 1.

**Table 1:** Demographic parameters of the study population (n=204) NIV: non-invasive ventilation; IMV: invasive mechanical ventilation; DM: diabetes mellitus; HTN; hypertension; COPD: chronic obstructive pulmonary disease; IHD: ischemic heart disease

Age, Mean ± SD (years)	55.7 ± 11.6
Gender, No. (%)	
Male	151 (74.0%)
Female	53 (26.0%)
Age groups, No. (%)	
Up to 30 years	8 (3.9%)
31 to 40 years	11 (5.4%)
41 to 50 years	43 (21.1%)
51 to 60 years	79 (38.7%)
61 to 70 years	49 (24.0%)
Above 70 years	14 (6.90%)
Comorbidities, No. (%)	
No comorbidity	125 (61.3%)
DM & HTN	31 (15.2%)
DM only	25 (12.3 %)
COPD	08 (4.0%)
HTN only	06 (2.9 %)
IHD	04 (2.0%)
Ventilatory Support, No. (%)	
NIV	126 (61.8 %)
IMV	78 (38.2 %)
Length of Stay, Mean ± SD (days)	6.2 ± 6.5
Up to 1 day	64 (31.4%)
2 to 3 days	32 (15.7%)
4 to 5 days	24 (11.8%)
More than 5 days	84 (41.2%)

The overall mortality was 77% (n=157). The difference in the gender-wise distribution (p=0.290) and mean age of survivors and non-survivors was insignificant (53.1±13.0 and 56.5±11.0 years, p=0.213). Mortality was significantly higher (93.6%) for patients on IMV as compared to those on NIV (66.7%) (p<0.001). Mortality among patients without co-morbidities was 75.2%. When compared to those without comorbidities, mortality rate for patients with at least one co-morbidity (79.7%), DM (80.0%), HTN (100%), both DM and HTN (87.1%), and chronic obstructive pulmonary disease (COPD) (75%) was not statistically significantly (p=0.452, 0.608, 0.163, 0.155, and 0.990, respectively). Patients older than 60 years had significantly higher mortality (87.3%) as compared to those younger than 60 years (72.3%) (p=0.019). Survivors stayed in the ICU for a significantly longer time as compared to non-survivors (8.9±8.9 versus 5.4±5.2 days, p=0.017). Staying for less than one day in the ICU was associated with higher mortality (85.9%) as compared to the LOS over one day (72.9 %), the difference being statistically significant (0.040) (Table 2).

**Table 2:** Comparison of variables among survivors and non-survivors * p-values for ‘At least one comorbidity’, ‘DM only’, ‘HTN only’, ‘DM &HTN’, and ‘COPD’ have been calculated taking ‘No comorbidity’ as reference. NIV: non-invasive ventilation; IMV: invasive mechanical ventilation; DM: diabetes mellitus; HTN: hypertension; COPD: chronic obstructive pulmonary disease

	Survivors (n=47)	Non-survivors (n=157)	P-value
Age, Mean ± SD	53.1±13.0	56.5±11.0	0.213
Ventilation Status, No. (%)			
NIV	42 (33.3%)	84 (66.7%)	<0.001
IMV	05 (6.4%)	73 (93.6%)	
Comorbidities No. (%)			
No comorbidity*	31 (24.8%)	94 (75.2%)	
At least one comorbidity	16 (20.3%)	63 (79.7%)	0.452
DM only	05 (20.0%)	20 (80.0%)	0.608
HTN only	0 (0%)	06 (100 %)	0.163
DM & HTN	04 (12.9%)	27 (87.1%)	0.155
COPD	02 (25%)	06 (75%)	0.990
Gender, No. (%)			
Male	32 (21.2%)	119 (78.8%)	0.290
Female	15 (28.3%)	38 (71.7%)	
Age group, No. (%)			
Up to 60 years	39 (27.7%)	102 (72.3%)	0.019
Over 60 years	08 (12.7%)	55 (87.3%)	
Length of stay, Mean ± SD	8.9±8.9	5.4±5.2	0.017
Up to one day	09 (14.1%)	55 (85.9%)	0.040
More than one day	38 (27.1%)	102 (72.9%)	

On logistic regression analysis, overall, the adjusted odds of mortality on IMV was 7.330 (95% CI 2.667 - 20.143) as compared to NIV (p<0.001). The odds of mortality for male patients were 1.104 times that of females (p=0.817). When compared to patients without comorbidities, none of the comorbid conditions affected mortality; DM (1.730, 95%CI 0.542 - 5.519, p=0.355), DM and HTN both (2.438, 95%CI 0.737 - 8.064, p=0.144), or COPD (0.688, 95%CI 0.111 - 4.264, p=0.688). Elderly (>60 years) patients were significantly more likely to die (2.607, 95%CI 1.063 - 6.394, p=0.036). A LOS of longer than 24 hours significantly decreased the likelihood of mortality (0.412, 95%CI 0.173 - 0.982, p=0.045) (Table 3).

**Table 3:** Multivariable analysis of risk factors for mortality AOR: adjusted odds ratio; CI: confidence interval; IMV: invasive mechanical ventilation; NIV: non-invasive ventilation; DM: diabetes mellitus; HTN: hypertension; COPD: chronic obstructive pulmonary disease; LOS: length of stay

	AOR	95% CI	P-value
Male/Female	1.104	0.477 - 2.556	0.817
IMV/NIV	7.330	2.667 - 20.143	<0.001
Over 60 years/under 60 years	2.607	1.063 - 6.394	0.036
LOS > 1 day / LOS < 1 day	0.412	0.173 - 0.982	0.045
DM/No DM	1.730	0.542 - 5.519	0.355
DM & HTN/No DM and HTN	2.438	0.737 - 8.064	0.144
COPD/No COPD	0.688	0.111 - 4.264	0.688

## Discussion

To date, there is no specific antiviral treatment for COVID-19. The backbone of management is to test, treat (isolate), and trace (quarantine). For most patients, hospitalization is not required. The main challenge has been many patients with rapidly progressive respiratory failure requiring supplemental oxygen, and some of them needing advanced respiratory support (NIV and IMV). To meet this challenge, every hospital expanded intensive care services and devised local guidelines amidst the pandemic.

The overall mortality was 77% in the present study. Being a developing country, the mortality rate is comparable to that reported by Richardson et al. from New York (75.6%) [7], Arentz et al. from Washington (67%) [22], and Yang et al. (61.5%) from China [10]. However, lower mortality rates have been reported by Mitra et al. from Canada (15.4%) [23], Lee et al. from Korea (20.4%) [24], and Grasselli et al. from Italy (26.0%) [8].

The reasons for the difference in survival compared to the developed countries could be due to the better availability of trained and dedicated staff for ICUs, the timely preparedness of the health system for the pandemic, sufficient resources, and the late phase of the pandemic in their regions. Moreover, in most of the above-mentioned studies, a significant number of patients were still receiving ICU care when the studies were published while in this study the outcome of all the admitted patients has been reported.

The mean age of patients admitted to the ICU was 55.7 ± 11.6 years. This contrasts with studies reported from the USA [22], Italy [8], and Spain [13], where the mean age of patients ranged from 63 to 73 years. Pakistan has only a 3.50 % population over the age of 65 years [25]. This might be the reason for the lower mean age of the patients in the present study.

Co-morbid conditions have been reported to contribute to the severity and worse outcomes in COVID-19. However, the distribution of comorbidities varies by region [4]. In the present study, DM was the leading co-morbid condition. In addition to 15.2% of patients who had both DM and HTN, 12.3 % had DM alone followed by COPD (4%) and HTN (2.9%). In other studies, HTN has been the most common co-morbid condition [8,11,13]. The high proportion of DM in this study may be due to the high prevalence of type 2 DM and pre-DM in Pakistan (16.98 % and 10.91%, respectively) [26]. This varied co-morbid profile also warrants further studies to better understand the impact of individual patient risk factors.

In the present study, 61% of patients did not have any co-morbid condition in contrast to that reported from Italy [8] and China [27], where 68% and 68.6% of patients, respectively had at least one co-morbid condition. The reason for the difference might be the younger age of our study population.

NIV has been the mainstay of treatment (61.8%) while 38.2% were treated by IMV. Yu et al. from Wuhan [27], Arentz et al. from Washington [22], and Grasselli et al. from Italy [8] have reported higher proportions of patients on IMV (37.6% versus 8.8%, 71% versus 19%, and 88% versus 11%, respectively). This might be explained by the fact that these studies were conducted during the early days of the pandemic when recommendations about the most appropriate modality of ventilation were not clear, and IMV was considered a more effective option. Moreover, there was a fear that NIV could generate an aerosol. It was quite late into the pandemic that NIV was advocated as the preferred alternative, especially for patients without significant co-morbidities [28].

A higher proportion of patients with DM only, HTN only, DM and HTN both, and COPD did not survive. But, when compared to those without any co-morbidity, the difference in mortality for any of the co-morbid conditions is not significant. This contrasts with published literature, where the presence of these co-morbidities increased the odds of mortality from COVID-19 [29]. However, a metanalysis (articles = 27, n=22,753) by Bajgain et al. concluded that the presence of one or more co-morbidity is not associated with a higher fatality rate [30].

Overall, the mortality was higher (93.6%) for those on IMV than those on NIV (66.7%). Treatment with NIV significantly increased the odds of survival (11.581, CI 3.307 - 40.553, p=<0.001). This is consistent with the mortality rate observed by Wang et al. and Yang et al. in China [10-11]. The high mortality with IMV patients could be due to inadequate intensive care services and lack of enough experience with treating COVID-19 related acute respiratory distress syndrome. Moreover, late shifting to the ICU at a stage where patients had developed multiorgan failure also added to the high mortality in this study.

When compared to younger patients (age less than 60 years), elderly patients had significantly higher mortality (OR 2.607, CI 1.063 - 6.394, p=0.036). Higher mortality among the elderly has been consistently reported [8,10].

The mean LOS was significantly longer for survivors (p=0.017). Similar observations were made by Grasselli et al. where the LOS for survivors was longer than non-survivors [8]. Mortality was higher (85.9% versus 72.9%) for those patients who stayed for less than one day in the ICU (p=0.040) regardless of the modality of ventilation. Longer stay (> one day) predicted lower mortality when adjusted for other variables. Though this finding is statistically significant, LOS less than 24 hours in the ICU might not have a causal relationship with mortality in COVID-19. To the best of our knowledge, mortality for less than 24 hours stay has not been reported. Most of the patients were brought by their relatives and friends late to the hospital and were directly admitted to the ICU in a moribund condition. Moreover, the shifting of critically ill hospitalized patients to the ICU was delayed due to the long waiting list. Multiple factors contributed to late presentation to the hospital and ICU. Pakistani society is known for illiteracy, superstition, and false beliefs. Many Pakistanis rejected the existence of the virus, blamed doctors for creating a myth of the virus, injecting poison and causing death, a conspiracy of western countries against religion, or believed that if you are affected, it is a punishment from God. The response of the government was also far from ideal. Tactics of using the police, rangers, and army for a selective lockdown of infected neighborhoods, escorting infected patients to isolation centers, the presence of guards at the cases’ residences, and repeated check on their family members at their homes regarding symptoms and test results, were tantamount to treating patients and their families like criminals. All these culminated in the social stigma attached to a COVID-19 diagnosis [18]. This led to the development of a reluctant attitude in the public to seek timely medical attention.

## Conclusions

The overall mortality in the present study was 77%. Age more than 60 years was an independent risk factor for higher mortality. Mortality was high for patients on IMV. The odds of mortality were not affected by DM, HTN, DM and HTN both, and COPD. Longer ICU stay, specifically, more than 24 hours, was associated with lower mortality but LOS less than 24 hours might not be associated with higher mortality.
